# Lost in Translation: Impact of Language Barriers and Facilitators on the Health Care of Asian Americans Living with HIV

**DOI:** 10.1007/s40615-023-01674-7

**Published:** 2023-06-12

**Authors:** Wei-Ti Chen, Wenxiu Sun, Feifei Huang, Cheng-Shi Shiu, Boram Kim, Jury Candelario, Lance Toma, Gilbert Wu, Judy Ah-Yune

**Affiliations:** 1 School of Nursing, University of California Los Angeles, Los Angeles, CA 90095, USA; 2 Shanghai Public Health Clinical Center, Fudan University, Shanghai, China; 3 School of Nursing, Fujian Medical University, Fuzhou, China; 4 National Taiwan University, Department of Social Work, Taipei, Taiwan; 5 APAIT- A division of Special Service for Groups, Inc., Los Angeles, CA, USA; 6 San Francisco Community Health Center, San Francisco, CA, USA; 7 Chinese-American Planning Council, Inc., New York, NY, USA

**Keywords:** Asian Americans, Language barriers, Facilitators, Health access, Communication, HIV-infected

## Abstract

Language barriers are major obstacles that Asian American immigrants face when accessing health care in the USA. This study was conducted to explore the impact of language barriers and facilitators on the health care of Asian Americans. Qualitative, in-depth interviews and quantitative surveys were conducted with 69 Asian Americans (Chinese, Filipino, Japanese, Malaysian, Indonesian, Vietnamese, and mixed Asian backgrounds) living with HIV (AALWH) in three urban areas (New York, San Francisco, and Los Angeles) in 2013 and from 2017 to 2020. The quantitative data indicate that language ability is negatively associated with stigma. Major themes emerged related to communication, including the impact of language barriers on HIV care and the positive impact of language facilitators—family members/friends, case managers, or interpreters—who can communicate with healthcare providers in the AALWH’s native language. Language barriers negatively impact access to HIV-related services and thus result in decreased adherence to antiretroviral therapy, increased unmet healthcare needs, and increased HIV-related stigma. Language facilitators enhanced the connection between AALWH and the healthcare system by facilitating their engagement with health care providers. Language barriers experienced by AALWH not only impact their healthcare decisions and treatment choices but also increase levels of external stigma which may influence the process of acculturation to the host country. Language facilitators and barriers to health services for AALWH represent a target for future interventions in this population.

## Introduction

Among all racial or ethnic groups in the United States (US), Asian Americans are the fastest-growing group [1] and in 2018, made up 6.7% of the total US population [2]. Asian Americans belong to diverse ethnic groups and reflect linguistic diversities. One study reported that more than 1000 languages/dialects are spoken by Asian Americans [3]. The Asian American population in the US has the highest proportion of residents who speak one or more languages at home other than English [3].

Approximately 30% of Asian Americans in the US have limited English proficiency (LEP) [1]. English proficiency significantly affects the acculturation process [2] when various challenges are faced such as separation from family and friends and adapting to a new culture, language, and lifestyle. English proficiency plays a vital role in dealing with a disease in the US healthcare system, especially when the system is new to the immigrant [3]. Language barriers impact access to healthcare and how healthcare services are understood [4]. Immigrants with LEP experience communication barriers that affect not only patient-provider relationships and reduce trust [4, 5] but also their health outcomes and quality of their medical visits [4]. Inefficient communication contributes to poor medical assessments, misdiagnoses, and delayed treatment [6]. Patients with LEP are more likely to have prolonged hospital stays [7], are at greater risk for hospital readmission [8], have fewer follow-up appointments [9], and have poorer medication adherence [10] and treatment compliance [4].

Acculturation, a process during which people leave their own culture for a host country culture, plays a key role in assimilating to the mainstream system [11]. According to Suinn-Lew (1987), if social friends’ ethnicities are the same as the study participants, in most cases, he/she will prefer to use their mother tongue to communicate [12]. In addition, if an individual prefers to use the native language to communicate, mostly maintain their LEP level [12]. One study has shown that people who stay in the US longer acculturate more to the US dominate culture [12]. In addition, if an individual arrives in the US at a young age and goes through the US education system, his/her LEP should be at a minimum [13].

For Asian immigrants living with HIV with LEP, stress during the acculturation process comes from a multitude of factors including discrimination from external sources (e.g., Asian hate), HIV disclosure struggles, health care access issues, and culture shocks [14, 15]. Several studies confirm that the acculturation process is associated with depression, stigma, and perceived stress [14, 16]. In particular, low or moderately acculturated Asian immigrants living with HIV present with depressive and stress symptoms [11]. In a recent study, perceived (felt) stigma can threaten a person’s own value [17]. Using Huang. et al.’s theoretical model (2020), we hypothesized that acculturation is negatively related to perceived stigma [14].

Asian Americans navigated the US healthcare system experiencing perceived stigma and discrimination especially Asian Americans with LEP [18]. Stigma that is experienced or anticipated can increase Asian Americans’ vulnerability to poor health outcomes, lead to lower healthcare access and engagement, and result in delays in seeking treatment and poor patient-HCP relationships [19, 20]. In addition, perceived stigma is also high when immigration status is required to disclosed in order to find a job or access social welfare [17], especially during the COVID pandemic with Asian hates [21, 22].

Asian Americans living with HIV (AALWH) are an especially vulnerable group [23–25], however, few studies have focused specifically on the impact of language barriers on HIV care. According to the US Centers for Disease Control (CDC), from 2010 to 2016, Asian Americans had the highest rate of HIV diagnoses (42%) compared to other racial/ethnic groups [26]. Additionally, Asian Americans present with low levels of HIV knowledge and have low HIV testing rates [25]. When diagnosed with HIV, Asian Americans are more likely to be at an advanced stage of AIDS and developed several opportunistic infections [24].

In this paper, we explore the impact of language barriers on AALWH as well as seek potential language facilitators. Findings from this study will guide development of interventions with AALWH that enhance communication between Asian Americans and their HCPs.

## Methods

### Study Design

This study not only designed to capture the richness of language barrier experiences in healthcare settings but also aimed to quantify language preferences and stigma levels in AALWH by using a mixed-methods study design. In total, we conducted 69 in-depth qualitative interviews with AALWH that were analyzed using content analysis. We used the content analysis to identify research themes (acculturation and stigma) and to identify the relationships of these two themes. Quantitative data were collected on language preference during the acculturation process and perceived and anticipated stigma via a survey.

### Study Settings and Sample

Community health clinics in New York, San Francisco, and Los Angeles provided approvals for research in their communities. Institutional Review Board (IRB) approvals were obtained from Yale University (HIC #1208010657) and from the University of California at Los Angeles (IRB#18–000025). Participants were recruited from the San Francisco Community Health Center (formally known as A&PI Wellness Center) in San Francisco, the Chinese-American Planning Council, Inc. (CPC), the Asian/Pacific Islander Coalition on HIV/AIDS Community Health Center (API-CHA) in New York City, and the Special Service for Groups-Access to Prevention Advocacy Intervention and Treatment (SSG-APAIT) in Los Angeles. Snowball and convenience sampling methods were used to identify AALWH who met the following inclusion criteria: (1) diagnosed with HIV, (2) self-identified as Asian American, and (3) 18 years of age or older.

A total of 69 AALWH participants from three urban centers were recruited. Multivariate logistic regression was used to test relationships between psychosocial factors, acculturation, and perceived stigma. The study had two data collection phases: (1) From January to June 2013, 50 AALWH participants were recruited in San Francisco, CA, and New York City, NY, and (2) From September 2017 to January 2020, 19 AALWH participants were recruited in Los Angeles, CA, and New York City, NY.

### Qualitative Data Collection

Prospective participants met with bilingual researchers. Researchers were trained on cross-cultural qualitative research methods and had PhD degrees in nursing and/or social work. After securing consent, 60–90 min, audio-recorded, in-depth, semi-structured interviews were conducted in the participant’s language of preference (Vietnamese, Cantonese, Mandarin, or English). Interview topics included experiences of acculturation, HIV diagnosis process, perceived stigma, HIV treatments, medical history, and language barriers and facilitators during medical visits. After two phases of in-depth interviews, data saturation was achieved.

### Quantitative Data Collection

A 30-min quantitative study survey with standardized measures was undertaken to gather data on demographics, social history, acculturation, and HIV stigma. The scales used in the survey have been used in many Asian populations and have shown good reliability and validity over time [27, 28]. Participants completed the survey through Audio Computer-Assisted Self-Interviews (ACASIs) or using Research Electronic Data Capture (REDCap). Small reimbursements ($15) were offered to compensate participants for their time and effort. To ensure the reliability of the study, we first pilot tested the semi-structured interview guide using two participants who were not included in the final study sample. Questions included: “Tell me when and why you decided to come to the United States?”; “What language do you prefer to speak at home, with your friends and at work? And why is that?”; “When and how did you know you were infected with HIV?”; “Tell me what happened when you needed to see the HIV doctor?”; and “Can you give me an example of when you feel that you were excluded or not welcome at a gathering? What happens?”; “Who helps you when you need to see the HIV doctor? What is the most helpful thing that you get from him/her/them?”. After the pilot participants expressed understanding of the interview questions, the in-depth interview was deployed to the study sample participants.

#### Demographics

Demographic data included age, sex, ethnic background, and immigrant status.

#### Suinn-Lew Asian Self-Identity Acculturation Scale

This 21-item scale measures six acculturation domains in Asian populations: language and cultural preference, interaction, generational identity, affinity for ethnic identity and pride, food preferences, and generation and geographic history [28]. In order to analyze correlations with stigma, we focused on six questions related to language ability from the full acculturation scale. Each item was scored from 0 to 4, and a total acculturation value was calculated by summing scores across the 6 items. The overall reliability of the Cronbach alpha in this instrument was 0.96 [13].

#### Stigma

The HIV Stigma Scale [27], a 40-item self-reported instrument that measures the degree of stigma experienced (*1* = strongly disagree, *2* = disagree, *3* = agree, and *4* = strongly agree), was used to evaluate perceived stigma. The total scores ranged from 40 to 160; higher scores indicated increased stigma. The subscale measured disclosure concerns, negative self-image, personal stigma, and concerns with public attitudes toward people with HIV. For this scale, the overall Cronbach’s alpha reliability was 0.95 [27].

### Analysis

All in-depth interviews were transcribed verbatim in the speaker’s language and coded and analyzed with qualitative content analysis [29] using the Atlas.ti 7.1.7 software (ATLAS.ti Scientific Software Development GmbH, Berlin, Germany). First, after completing the in-depth interviews, transcriptions without individual identifiers were shared with a few study participants for transcription validation. Second, the study team looked for concept categories and code trees related to language barriers. Third, two researchers inspected the transcriptions individually and assigned codes based on themes. To ensure coding inter-rater reliability, two researchers coded the first 5 transcriptions and checked the consistency of coding. The study team discussed the coding discrepancies and achieved consensus. Representative quotations related to language barriers and stigma experiences were then selected and, where necessary, translated into English.

Quantitative data analyses were conducted using SPSS 24.0 (IBM, Chicago, IL). The continuous variables were expressed as mean and standard deviation (SD); categorical variables were expressed as percentages. We conducted multivariate logistic regression analysis to identify and check associations between language acculturation and stigma. Independent variables with *p* < 0.05 was considered significant. Missing data were replaced by the average of observed data.

## Results

Demographic data including participants’ language preference and social friends’ ethnicities are presented in Table 1. The age range of the study participants was 31 to 72 years (mean = 51 years, SD =10.4) and approximately 78% of the participants were male. Participants had various ethnic backgrounds: Chinese, Filipino, Japanese, Malaysian, Indonesian, Vietnamese, and mixed Asian. The majority reported that they were US immigrants and more than three-quarters of the participants (78.3%) were raised in Asia.

### Quantitative Analysis

The results of multivariate logistic regression are shown in Table 2. Different demographic (age, sex, ethnicity, immigrant), language acculturation, and stigma were entered in stepwise regression. The results of multivariate logistic regression analysis (*F=*3.17, *p=*0.009) suggest acculturation levels were negatively affected by the respondents’ stigma (*β*= −0.541, *p =*0.002).

### Qualitative Analysis

#### Impact of Language Barriers on HIV Health Care

Qualitative data analysis revealed several themes relating to the impact of language barriers on HIV health care: impeded access to HIV healthcare services, decreased adherence to antiretroviral therapy (ART), increased unmet healthcare needs, and increased stigma experienced in healthcare settings (Table 3).

#### Impeded Access to HIV Healthcare Services

As immigrants, the participants felt unfamiliar with local healthcare systems, which sometimes led to delayed HIV testing. For example, one 69-year-old male stated:

When I came here (San Francisco), the health system was new to me… I couldn’t speak English… I had no idea that I could get those services here. I could have come to check [my] HIV status two years earlier. In my case, I can get treatment, but I didn’t know, so it was very late for me to find out I had this (HIV).

Similarly, another male participant (53 years old) expressed that, “As an Asian American, the difficulty of medical care in the US was [the] language barrier.”

#### Decreased ART Adherence

Participants with LEP had difficulty understanding their HIV diagnosis, communicating with HCPs about their disease, and understanding prescriptions. These communication problems decreased the AALWH’s ability to follow health care instructions and interfered with their ART adherence, which resulted in delays starting ART and poor ART adherence. On male participant (51 years old) said:

When I did the physical examination, the doctor only spoke English and there was no translation. I didn’t get my test results for that visit. Then I got a letter with an underlined word of “HIV.” I didn’t know what the letters meant. Two years later, I did another physical examination and was told that I am HIV-positive. It was almost a 2-year delay.

A female participant (49 years old) had a similar experience:

When I did the medical test, my doctor told me I have HIV and he gave me the antiretroviral medicine for 3 months. Because I didn’t understand English and didn’t know what HIV was, I did not take the medicine. Later, when I felt that my health was in trouble, especially some neuropathy in my eyes and headaches, I went to the hospital again and found out the HIV diagnosis.

#### Increased Unmet Healthcare Needs

One of the participants expressed problems while hospitalized due to the language barrier. One 72-year-old female shared:

During hospitalization, I got steak, chicken, and some-thing like that. I am a vegetarian but can’t speak English. So, I didn’t know how to tell them what I can eat. I didn’t eat anything and felt I would starve to death.

Even though some bilingual participants expressed that they were able to communicate in English, they had trouble understanding medical terminology. One 61-year-old male said:

I think my spoken English is OK. But for some professional issues, I still need a translator, like in the pharmacy. Sometimes I don’t understand the doctor. But I know he doesn’t want to kill me. He is trying to help me. I just don’t know what he wants me to do.

#### Increased Stigma in Healthcare Settings

A few participants described their experiences of perceived stigma by HCPs were due to language barriers. One 72-year-old male stated:

There is one time that I went to see the doctor and waited there after check-in. I waited there for nearly 3 hours. Everyone had seen the doctor, but not me. I went to ask the doctor. But he ignored me. So, I continued to wait until 4 p.m. Then, the receptionist told me that the doctor had no time to see me. Later, my case manager told me [that] because of my limited English proficiency, it would better to find my own interpreter. So, after that, I hired my own interpreter when I went for the follow-up visits.

#### One 59-year-old female shared:

When I did my mammogram, my translator couldn’t come in with me. So, I went in by myself. There was a big machine. A Spanish technician told me to do some things, but I couldn’t understand. Then she was very fierce and pushed me aside. She also scolded me in English. It is probably because I had HIV and she looked down on me.

Because of LEP, some participants only see a HCP with the same language background. However, others preferred not to seek an HCP with a similar background in an effort to avoid anticipated stigma. One 61-year-old male explained:

I never want to see a Chinese-speaking doctor. I have HIV, and I don’t want to see a Chinese doctor. We are Chinese, but you never know (whether) the doctor might know someone that you know. Doctors from other backgrounds… [who] know I have HIV… will be less likely to disclose my status.

### Facilitators that Could Enhance HIV Care

Participants shared several ways that their HIV care could be facilitated. These included getting assistance from case managers, having family members or friends accompany them, and only making appointments with same-language HCPs.

#### Assistance from Case Managers

Most of participants expressed their gratitude for case managers. One 56-year-old male said:

When I purchased life insurance, I was asked to do a blood test and found out that I was HIV infected. The salesperson in the insurance company introduced me to the Chinese-American Planning Council. The case managers in CPC helped me contact HIV doctors, accompanied me to see the doctor, and helped me apply for social welfare. It has been 20 years till now. I can’t speak English, and I need them to translate. They not only translate the doctor’s words, but also told me how to take medicine and what things I need to pay more attention to.

Some case managers not only physically accompanied the participant on HCP’s appointments, but also helped via phone-based remote translations. One 53-year-old male stated, “Sometimes the case manager can’t make the visit with me; then I just call and she can translate instantly.”

However, while many non-government organizations (NGOs) provide case managers to facilitate doctors’ appointments, some hesitate to request them for translation, especially if there is the potential of disclosing one’s HIV status. One 38-year-old female noted such a breach of confidentiality:

Every time I went for a follow-up visit, the case manager would come with me. They have done a good job. [However,] when she introduced herself as an HIV case manager, I felt that she was saying that I am an HIV-infected patient. I felt a little uncomfortable. One time, two of us [patients] needed to see doctors in the same department. When I saw the other patient, I blushed and didn’t dare to say hello. I know the case manager’s work, and the other client also knew. That means both of us are HIV-infected. That was really awkward.

#### Assistance from Family Members or Friends

Participants who cannot communicate in English often seek support from family members. One 52-year-old female stated, “The first time that I saw the doctor, I was very depressed without helI just could not talk…so now, I ask my sister and my sons to help me.”

Several participants reported that family members/friends needed to know their HIV status before they could ask them to facilitate their health care. One 53-year-old male said, “My boyfriend is a Vietnamese Chinese. I told him I have HIV and he was okay with my situation. Therefore, sometimes he translates for me when I have a doctor’s visit.”

#### Locating HCPs Who Can Communicate in the Same Language of Preference

Participants described how they tried to find an HCP who could communicate in their own language to avoid miscommunication. One 51-year-old male explained:

As Asians, we need support, we need care, and we need attention. Of course, you can go to a different group for health care, but it is the best thing that we had the same ethnic background. I did search for a while. I was searching for the right doctor, and I tried; finally, I found someone. He told me that he is a Vietnamese doctor. The best thing is…he is an Asian and has the same color as me. He is also a doctor and speaks the language. Of course, he understood the culture better. We had the same mentality, the vision, and our mother tongue. When I talk to my doctor in the same language, that feels much better. We had more connection. It is very important for me to know that the person who provides care can really connect to me.

## Discussion

Language barriers significantly impact AALWH patients’ adherence to medication and quality of care [19]. The present study highlights the vulnerabilities of Asian Americans with LEP in their use of healthcare services. While language barriers can also be an issue for those from other ethnic backgrounds, AALWH face additional barriers from stigma stemming from both racism and stereotyping [23–25]. In this study, we explore the impact of language barriers on HIV care among AALWH and identify potential language facilitators to enhance care.

The ability to communicate in English plays a vital role in successfully engaging in healthcare services in the US [4]. Most of the participants in this study were immigrants to the US and more than half of them reported having LEM-ore than two-thirds preferred to use their native language in everyday activities, supporting our hypothesis that language barriers are a common experience among AALWH, not only in HIV-related care but also in other health-seeking behaviors. AALWH with LEP in this study received suboptimal care, which is similar to findings of other studies [24, 25].

The impacts of language barriers on HIV care are many for immigrants. Firstly, familiarity with the US healthcare system is lacking. Many AALWH have already developed AIDS before seeking care due to not knowing where to go for HIV testing or not understanding what HIV is, or its high-risk behaviors. In many cases, a confirmed diagnosis was received only after developing full-blown AIDS, a finding supported by other studies [24]. Secondly, the quality of care was significantly decreased due to language barriers. When AALWH are unable to express their needs, their own health may deteriorate and they will also pose a greater risk for transmitting HIV to others. Thirdly, communication difficulties can reduce trust between a patient and their HCP, decrease cooperation and their adherence to medical advice, cause missed appointments, and result in poorer health outcomes [30]. One study participant was ignored by the healthcare team, waiting for three hours and ended up not receiving any care. Due to language barrier and their HIV status, they were stigmatized during the health care seeking process. Lastly, as shown in both the quantitative and qualitative results of this project, language barriers increase AALWH’s perceived stigma and are associated with level of acculturation.

Our findings highlight the stigma that AALWH with LEP face in HIV health care. Regression analysis identified a strong relationship between stigma and language ability, which is an important part of acculturation. Consistent with previous studies on immigrants, individuals who came to the US at a younger age, had longer stays in the US, had more non-Asians friends, and had better English proficiency, experienced less perceived discrimination in this study and others [19, 31]. In contrast, immigrants with high levels of perceived stigma tended to socialize with Asian friends, had less access to health care resources, and maintained limited social connections to the greater society - findings that are echoed in other studies [2, 19]. In addition, the stigma perceived by AALWH with LEP not only leads to dissatisfaction with health care but also influences how health care services are sought in the future. Other studies have shown that individuals prefer culturally and linguistically concordant HCPs [5]. However, the situation is more complex among AALWH. In this study, some participants preferred not to choose HCPs with similar backgrounds due to the perceived increased risk of HIV disclosure and discrimination. Compared to other ethnicities, AALWH experience higher levels of perceived HIV-related stigma which affects their healthcare-seeking behaviors [19]. In traditional Asian cultural contexts, HIV is synonymous with homosexuality, drugs, and extramarital relationships, all of which are deeply stigmatized [32] and creates a closed, intolerant community that leads to fear of HIV, stigmatization, and exclusion of AALWH from mainstream society [23]. Thus, stigma is an essential factor to consider in planning future interventions to enhance AALWH’s engagement with healthcare systems.

Our findings indicate community agencies are essential in connecting AALWH to the healthcare system in the US. Case managers are the most frequent and important liaisons between AALWH and HCPs. Case managers are readily available, easy to access, familiar with the unique challenges facing each client, and are usually free of charge. In the US, several community agencies provide case management services to AALWH. These services, including medical care assistance, social welfare applications, and language services, are cost-free and available upon request. Case managers also encourage adherence to medical treatments and coordinate a range of services needed to maintain physical and mental health [23]. Conversely, in order to avoid potential disclosure of their HIV status, some participants prefer not to use them, a concern shared by other populations [33] that highlights the importance of privacy and confidentiality in the context of health care.

Similar to other studies on LEP [34], family and friends can serve as translators to overcome language barriers. In most situations, family or friends are the most accessible and convenient resource for language translation [35]. In this study, family or friends are a source of support for AALWH but, for those AALWH who have yet to disclosure their HIV status, the use of family or friends for support is not encouraged [36, 37]. Several issues arise from using family members/friends as language translators. Firstly, the quality of interpreting may be compromised [38], due to medical jargon and translational errors with harmful consequences and possible omissions of care may occur [7]. Secondly, providers asking AALWH sensitive questions via their children can interfere with family roles [39] and either false information or misinterpretations can occur that affect their disease management. Thirdly, relying on family/friends to translate creates breaches in confidentiality [40]. Thus, to safeguard against violations of privacy, refraining from requesting family members or friends to serve as language translators for AALWH is always preferred, and professional translator services are recommended.

This study has several limitations. Firstly, participants in this study were recruited from urban centers where culturally-relevant services and resources are more readily available. Therefore, this study may not represent all AALWH in the US and its results may be limited in generalizability. Secondly, selection bias may be present since participants may have had an interest and awareness of their language needs vis-à-vis healthcare engagement. Thirdly, we were limited in recruiting participants who spoke certain Asian languages (English, Mandarin, and Cantonese), which may have biased the results. Fourthly, the long timeframe of the study and our sampling methods may have introduced sampling bias. In addition, the majority of participants were males, and therefore, results may not be generalizable to females. Lastly, the small sample size of AALWH used in our correlational analysis of acculturation and stigma potentially limited the interpretation of this association.

## Conclusion

This study shows AALWH face language barriers when accessing and receiving HIV care in the US. In addition, results indicate those who are acculturated to the US experience less perceived stigma. Lack of language ability may discourage AALWH from seeking health care in the first place through its impacts on access to healthcare services and perceived potential stigma from healthcare settings, resulting in poorer medication adherence and unmet health needs. Potential language facilitators—family members/friends, case managers, interpreters, or healthcare providers with congruent cultural backgrounds—may enhance quality of care and engagement in healthcare services. Based on this study, a targeted and culturally sensitive intervention should be developed to enhance healthcare experiences in this population.

## Figures and Tables

**Table 1:** Demographic data

	N=69 (%)
Age (years)	
Mean=51, SD=10.4	
Range 31–72	
31–50	30 (43.5)
51–72	39 (56.5)
Sex	
Male	54 (78.3)
Female	15 (21.7)
Ethnicity	
Chinese	35 (50.7)
Philippine	11 (15.9)
Vietnamese	7 (10.1)
Malaysian	5 (7.2)
Japanese	3 (4.3)
Indonesian	1 (1.4)
Other	7 (10.1)
Education	
11^th^ grade or less	33 (47.8)
High school or GED	20 (29.0)
2 years of college/technical school training	10 (14.5)
College (Bachelor’s)	5 (7.2)
Master degree/doctorate/medical degree/law degree	1 (1.4)
Working status	
Not working	49 (71.0)
Part time	10 (14.5)
Full time	10 (14.5)
Immigrant (yes/no)	
Yes	63 (91.3)
Language preference (%)	
Asian language	47 (68.1)
Equally good	8 (11.6)
Mostly English	14 (20.3)
Friend and peers’ ethnicity	
Mostly Asians	58 (84.1)
Equally with other ethnicity	5 (7.2)
Mostly non-Asians	6 (8.7)
Length of stay in the US (mean/years)	21.3 (2.1)
Age at arrival (mean)	29.7 (11.5)
Acculturation level (language)	7.8 (7.9)
Stigma (mean)	52.2 (19.3)

**Table 2:** The multivariate logistic regression between demographic, language acculturation, and stigma

Variables	Stigma					
	B	β	t	p	F	Adjusted R^2^
Age	−0.078	−0.044	−0.293	0.770	3.17**	0.161
Sex	4.943	0.111	0.957	0.342		
Ethnicity	1.122	0.179	1.499	0.139		
Immigrant	−13.677	−0.21	−1.417	0.161		
Length of stay in the US	0.02	0.013	0.079	0.937		
Acculturation (language)	−1.273	−0.541	−3.188	0.002**		

** *P* ≤0.05.

**Table 3:** Themes and sub-themes from participants’ in-depth interview transcriptions

Themes	Sub-themes
Impact of language barriers on HIV health care	Impeding access to HIV healthcare services Decreased antiretroviral therapy adherence Increased unmet healthcare needs Increased stigma in healthcare settings
Language facilitators that could enhance HIV care	Assistance from case managers Assistance from family members or friends Looking for an HCP who can communicate in their mother tongue

## References

[1] Haley JM, Zuckerman S, Rao NN, Karpman M, Stern A. Many Asian American and native Hawaiian/Pacific islander aduts may face health care access challenges related to limited english proficiency. https://www.urban.org/sites/default/files/2022-12/Many%20AANHPI%20Adults%20May%20Face%20Health%20Care%20Access%20Challenges%20Related%20to%20Limited%20English%20Proficiency.pdf.

[2] Martinez-Donate AP, Does acculturative stress influence immigrant sexual HIV risk and HIV testing behavior? Evidence from a survey of male Mexican migrants. J Racial Ethn Health Disparities. 2018;5(4):798–807. 28840518 10.1007/s40615-017-0425-2 PMC5826773

[3] Chen WT. Chinese female immigrants english-speaking ability and breast and cervical cancer early detection practices in the New York metropolitan area. Asian Pac J Cancer Prev. 2013;14(2):733–8. 23621228 10.7314/apjcp.2013.14.2.733 PMC5345995

[4] Jang Y, Kim MT. Limited English proficiency and health service use in Asian Americans. J Immigr Minor Health. 2019;21(2):264–70. 29797103 10.1007/s10903-018-0763-0 PMC6252148

[5] Yeheskel A, Rawal S. Exploring the ‘patient experience’ of individuals with limited english proficiency: a scoping review. J Immigr Minor Health. 2019;21(4):853–78. 30203377 10.1007/s10903-018-0816-4

[6] de Moissac D, Bowen S. Impact of language barriers on quality of care and patient safety for official language minority francophones in Canada. J Patient Exp. 2019;6(1):24–32. 31236448 10.1177/2374373518769008 PMC6572938

[7] Patel AT, Length of stay for patients with limited english proficiency in pediatric urgent care. Clin Pediatr (Phila). 2020;59(4–5):421–8. 31994413 10.1177/0009922820902439

[8] Wilbur MB, Unplanned 30-day hospital readmission as a quality measure in gynecologic oncology. Gynecol Oncol. 2016;143(3):604–10. 27665313 10.1016/j.ygyno.2016.09.020

[9] Gallagher RA, Unscheduled return visits to the emergency department: the impact of language. Pediatr Emerg Care. 2013;29(5):579–83. 23603647 10.1097/PEC.0b013e31828e62f4

[10] Squires A, Assessing the influence of patient language preference on 30 day hospital readmission risk from home health care: a retrospective analysis. Int J Nurs Stud. 2022;125:104093. 34710627 10.1016/j.ijnurstu.2021.104093 PMC9282680

[11] Chen WT, Acculturation and perceived stress in HIV+ immigrants: depression symptomatology in Asian and Pacific Islanders. AIDS Care. 2014;26(12):1581–5. 25059642 10.1080/09540121.2014.936816 PMC4188794

[12] Suinn RM. Measurement of Acculturation of Asian Americans. Asian Am Pac Isl J Health. 1998;6(1):7–12. 11567398

[13] Barry DT. Development of a new scale for measuring acculturation: the East Asian Acculturation Measure (EAAM). J Immigr Health. 2001;3(4):193–7. 16228786 10.1023/A:1012227611547

[14] Huang F, Acculturation, HIV-related stigma, stress, and patient-healthcare provider relationships among HIV-infected Asian Americans: a path analysis. J Immigr Minor Health. 2020;22(6):1217–24. 32789735 10.1007/s10903-020-01068-5 PMC7424136

[15] Tang K, Chen WT. HIV and religion in HIV-infected Asians and their families: a qualitative study. Appl Nurs Res. 2018;44:18–24. 30389055 10.1016/j.apnr.2018.09.003 PMC6220712

[16] Chen WT, Revising the American dream: how Asian immigrants adjust after an HIV diagnosis. J Adv Nurs. 2015;71(8):1914–25. 25740206 10.1111/jan.12645 PMC4503482

[17] Becker TD, The impact of China-to-US immigration on structural and cultural determinants of HIV-related stigma: implications for HIV care of Chinese immigrants. Ethn Health. 2022;27(3):509–28. 32668975 10.1080/13557858.2020.1791316 PMC7854980

[18] Sen S, Aguilar J, Petty M. An ecological framework for understanding HIV- and AIDS-related stigma among Asian American and Pacific Islander men who have sex with men living in the USA. Cult Health Sex. 2021;23(1):85–97. 32031498 10.1080/13691058.2019.1690164

[19] Clough J, Lee S, Chae DH. Barriers to health care among Asian immigrants in the United States: a traditional review. J Health Care Poor Underserved. 2013;24(1):384–403. 23377740 10.1353/hpu.2013.0019

[20] McMurtry CL, Discrimination in the United States: experiences of Asian Americans. Health Serv Res. 2019;54(Suppl 2(Suppl 2)):1419–30. 31657465 10.1111/1475-6773.13225 PMC6864377

[21] Gover AR, Harper SB, Langton L. Anti-Asian Hate crime during the COVID-19 pandemic: exploring the reproduction of inequality. Am J Crim Justice. 2020:1–21. 10.1007/s12103-020-09545-1 PMC7364747 32837171

[22] Macaraan WER. The notion of Kapwa amid Asian hate. J Public Health (Oxf). 2021;44(3):e421–2. 10.1093/pubmed/fdab268 PMC8344461 34240214

[23] Pichetsurnthorn P, HIV care in Asian and Pacific Islanders in Kansas. J Int Assoc Provid AIDS Care. 2019;18:2325958218821650. 10.1177/2325958218821650 PMC6748497 30798680

[24] Russ LW, Examining barriers to care: provider and client perspectives on the stigmatization of HIV-positive Asian Americans with and without viral hepatitis co-infection. AIDS Care. 2012;24(10):1302–7. 22440043 10.1080/09540121.2012.658756

[25] Sen S, HIV Knowledge, risk behavior, stigma, and their impact on HIV testing among Asian American and Pacific Islanders: a review of literature. Soc Work Public Health. 2017;32(1):11–29. 27410387 10.1080/19371918.2016.1173612

[26] Center for Disease Control and Prevention., HIV among Asian Americans. 2020, ,Center for Disease Control and Prevention: Atlanta, GA.

[27] Berger BE, Ferrans CE, Lashley FR. Measuring stigma in people with HIV: psychometric assessment of the HIV stigma scale. Res Nurs Health. 2001;24(6):518–29. 11746080 10.1002/nur.10011

[28] Suinn RM, The Suinn-Lew Asian self-identity acculturation scale: an initial report. Educ Psychol Meas. 1987;4:401–7.

[29] Hsieh HF, Shannon SE. Three approaches to qualitative content analysis. Qual Health Res. 2005;15(9):1277–88. 16204405 10.1177/1049732305276687

[30] Sabato TM. A comprehensive approach to risk reduction for Asian and Pacific Islander American women with HIV/AIDS. J Transcult Nurs. 2014;25(3):307–13. 24570381 10.1177/1043659614523452

[31] Jung H, Stigmatizing beliefs about depression in diverse ethnic groups of Asian Americans. Community Ment Health J. 2020;56(1):79–87. 31578672 10.1007/s10597-019-00481-x

[32] Vlassoff C, Ali F. HIV-related stigma among South Asians in Toronto. Ethn Health. 2011;16(1):25–42. 21259140 10.1080/13557858.2010.523456

[33] Dapaah JM, Senah KA. HIV/AIDS clients, privacy and confidentiality; the case of two health centres in the Ashanti Region of Ghana. BMC Med Ethics. 2016;17(1):41. 27422295 10.1186/s12910-016-0123-3 PMC4947355

[34] Jaeger FN, The migration-related language barrier and professional interpreter use in primary health care in Switzerland. BMC Health Serv Res. 2019;19(1):429. 31248420 10.1186/s12913-019-4164-4 PMC6598246

[35] Gray B, Hilder J, Donaldson H. Why do we not use trained interpreters for all patients with limited English proficiency? Is there a place for using family members? Aust J Prim Health. 2011;17(3):240–9. 21896260 10.1071/PY10075

[36] Czapka EA, Gerwing J, Sagbakken M. Invisible rights: barriers and facilitators to access and use of interpreter services in health care settings by Polish migrants in Norway. Scand J Public Health. 2019;47(7):755–64. 30345877 10.1177/1403494818807551

[37] Lee JS, Hospital discharge preparedness for patients with limited English proficiency: a mixed methods study of bedside interpreter-phones. Patient Educ Couns. 2018;101(1):25–32. 28774652 10.1016/j.pec.2017.07.026 PMC5732033

[38] Gerrish K, Bridging the language barrier: the use of interpreters in primary care nursing. Health Soc Care Community. 2004;12(5):407–13. 15373819 10.1111/j.1365-2524.2004.00510.x

[39] Cohen S, Moran-Ellis J, Smaje C. Children as informal interpreters in GP consultations: pragmatics and ideology. Sociol Health Illn. 1999;21(2):163–86.

[40] Patriksson K, Health care professional’s communication through an interpreter where language barriers exist in neonatal care: a national study. BMC Health Serv Res. 2019;19(1):586. 31426785 10.1186/s12913-019-4428-z PMC6701045

